# Discussing HPV with oropharyngeal cancer patients: A cross-sectional survey of attitudes in health professionals

**DOI:** 10.1016/j.oraloncology.2017.03.014

**Published:** 2017-05

**Authors:** Rachael H. Dodd, Alice S. Forster, Jo Waller, Laura A.V. Marlow

**Affiliations:** Cancer Communication & Screening Group, Research Department of Behavioural Science and Health, UCL, Gower Street, WC1E6BT, United Kingdom

**Keywords:** Head and neck cancer, Human papillomavirus, Attitudes, Health professionals, Knowledge

## Abstract

**Background:**

Human papillomavirus (HPV) is now known to cause a significant proportion of head and neck cancers (HNC). Qualitative research has shown that some health professionals find it difficult to discuss HPV with patients due to its sexually transmitted nature, and have concerns about their own knowledge of the virus. We used a survey to quantify attitudes towards discussing HPV among HNC health professionals.

**Methods:**

We carried out a cross-sectional survey of HNC health professionals (n = 260) in the UK and Ireland, assessing participants’ knowledge of HPV, their experiences of and attitudes towards discussing HPV with patients, and their willingness to discuss HPV with their patients in the future.

**Results:**

Overall, health professionals had good knowledge of HPV (mean score: 9.97 out of 12). Oncologists had significantly greater knowledge than specialist nurses, speech and language therapists and ‘other’ health professionals. Most were willing to discuss HPV with patients (mean = 4.3 out of 5). Willingness to discuss HPV in the future was associated with higher HPV knowledge (r = 0.35, p < 0.001), fewer negative and more positive attitudes towards discussing HPV (r = −0.23 and r = 0.20 respectively, both p < 0.001), fewer personal barriers (r = −0.49, p < 0.001) and greater confidence (r = 0.58, p < 0.001).

**Conclusion:**

Knowledge, experiences and attitudes to discussing HPV varied across HNC health professionals. Addressing gaps in health professionals’ HPV knowledge and improving their confidence in discussing HPV with patients may increase their willingness to have such conversations. This may help minimise the negative psychosocial consequences of an HPV diagnosis in this patient group.

## Highlights

• Knowledge of HPV was good among HNC HCPs, but there were some gaps in knowledge.
• Health professionals mainly had positive attitudes to discussing HPV.
• Most HCPs demonstrated willingness to discuss HPV with their patients in the future.
• Confidence and less personal barriers predicted willingness to discuss HPV.

## Introduction

Overwhelming evidence has demonstrated that human papillomavirus (HPV) plays a causal role in some oropharyngeal squamous cell carcinomas (OSCC) [1], in addition to cervical, anal, penile, vulva and vaginal cancers [2]. In the US, HPV is detected in two-thirds of oropharyngeal cancers [3]. Studies have shown HPV-OSCC is prevalent worldwide and incidence rates are expected to rise [4], with numbers in the US predicted to exceed cervical cancer cases by 2020 if the current trend continues [5]. Research has shown both oral sex and open mouth kissing are associated with acquisition of oral HPV infection [6].

Consistent with findings from the cervical cancer literature [7], [8], studies conducted with head and neck cancer (HNC) health professionals have identified psychological and communication challenges associated with the discussion of HPV, primarily due to its sexually transmitted aetiology [9], [10]. In a UK-based study, some health professionals also expressed concern that their knowledge about the role of HPV in HNC was limited and that this made it difficult for them to respond to the questions and concerns of patients [9].

There is wide variation in awareness of the association between HPV and HNC among health professionals involved in the diagnosis and treatment of HNC. A recent review [11] has shown that knowledge of the association between HPV and HNC ranges from 26 to 88% in dentists [12], [13] and 34 to 91% in other medical professionals [14], [15]. Variation has also been shown within oral health professionals in the US, ranging from a complete lack of knowledge, to understanding of some details, but none had high levels of knowledge [10]. Participants in the study wanted to improve their knowledge and have training to aid in communicating with patients and this may be important in implementing consistent messages about HPV.

Previous studies suggest that greater knowledge influences communication related to HPV, for example health professionals with greater knowledge about HPV and the HPV vaccination, are more likely to recommend the vaccine [16] and self-rated knowledge about HPV vaccination has been shown to be an important determinant of willingness to recommend the HPV vaccination among doctors [17]. Some dental professionals say that they need more information and materials which could help them facilitate conversations about HPV-OSCC with their patients [10], but this has not been investigated with HNC health professionals.

This study was carried out in the UK and Ireland, and aimed to assess knowledge of HPV, experiences, and attitudes to discussing HPV across different groups of health professionals involved in the treatment of HNC. We also examined how these factors were associated with willingness to discuss HPV with patients in the future.

## Methods

### Participants

Health professionals in roles working directly with HNC patients (surgeons, oncologists, specialist nurses and allied health professionals) in the UK or Ireland completed an online or paper survey. A number of methods of recruitment were used. Professional organisations (e.g. National Cancer Research Institute, National Cancer Intelligence Network, NHS Cancer Networks [18]), and existing contacts in the NHS and from previous research studies were contacted. Delegate lists from relevant conferences were used to help provide the names of HNC health professionals and email addresses were sourced for these names where possible (n = 246). Health professionals were also recruited at three HNC conference days where data were collected using paper questionnaires (n = 160). Further information about the methods of recruitment is provided as supplementary material.

We also asked participants who had already taken part in the study to contact others who they thought might also be eligible and willing to take part; a technique known as ‘snowballing’ [19]. Where possible, reminders were sent to those who received the online link to the survey two and four weeks after the initial email.

Because of this method of recruitment, the response rate is unknown. The size of the cancer networks and organisations was unknown and some health professionals may have received the survey from multiple sources. A sample size calculation with α = 0.05 and a power of 0.8, suggested that 220 participants were needed to detect differences in knowledge across the health professional groups with a medium effect size of r = 0.3.

### Ethical approval

This study was approved by the UCL Research Ethics Committee, reference 4577/003.

### Measures

The survey assessed experience of discussing HPV, knowledge of HPV, attitudes to discussing HPV and willingness to discuss HPV in the future. Socio-demographic factors were also assessed. Most of the items were developed from a previous qualitative study [9] or previous studies exploring attitudes and knowledge relating to HPV [7], [14]. The survey is included as supplementary material.

Experience of discussing HPV: Four items assessed health professionals’ experiences of informing HNC patients about HPV: ‘Thinking about the patients you treat with HPV-related head and neck cancer, how many of them have you told that their cancer was caused by HPV?’ (all; most; some; none) and ‘If you have informed a patient about their HPV status, have you discussed HPV in detail with a patient?’ (yes; no; not sure). Health professionals were also asked, ‘If you discuss HPV during consultations, who usually initiates the discussion?’ (myself; patient; sometimes me, sometimes the patient; other; not applicable).

Knowledge of HPV: Health professionals were asked ‘Have you ever heard of HPV?’ (yes; no; don’t know). Health professionals were also asked to respond ‘true’, ‘false’ or ‘don’t know’ to twelve knowledge items. Seven of these were from a validated measure of HPV knowledge [20] and five were specifically about HPV and HNC (adapted from a previous study [14]). A total score for knowledge was calculated by summing the number of correct responses to give a maximum possible score of 12 (Cronbach’s α = 0.62).

Twenty-five items assessed health professionals’ attitudes towards discussing HPV with patients (see supplementary information). Responses to these questions were on a 5-point Likert scale (strongly disagree, disagree, neither agree nor disagree, agree, strongly agree). These items were developed based on the findings from the existing literature [7], [9], [14]. Principal component analysis (PCA) yielded five factors: confidence in discussing HPV (5 items; α = 0.89), negative attitudes to discussing HPV (5 items; α = 0.75), positive attitudes to discussing HPV (5 items; α = 0.76), personal barriers to discussing HPV (6 items; α = 0.78) and needing more information (4 items; α = 0.64). Two items in the factor ‘personal barriers to discussing HPV’ were reverse scored before running further analysis (see supplementary information). Participants with missing data on any of the 25 items were excluded from this analysis (n = 6).

Willingness to discuss HPV in future: Health professionals were asked, ‘Generally, how willing are you to discuss HPV with your patients in the future?’ (not at all willing; not very willing; neither willing or unwilling; somewhat willing; very willing).

Socio-demographic and professional background items assessed age, sex, profession (surgeon, oncologist, specialist nurse, speech and language therapist, other), number of years in profession, whether they had trained in the UK and their main place of work (hospital, hospice, rehabilitation centre, other). Participants were also asked, ‘Have you ever looked for any information on human papillomavirus (HPV) and head and neck cancer?’ and ‘if yes, where have you looked?’: internet, medical journals, other colleagues, conferences, professional organisations, media, other.

### Analysis

Chi-square tests were used to compare responses to individual knowledge items across health professional groups and ANOVA was used to compare total knowledge score between groups.

ANOVA was used to compare each attitudinal factor from the PCA across health professional groups. Pearson’s correlations were run to explore the relationships between the attitudinal factors, willingness to discuss HPV in the future, years practising in their profession and knowledge. A binary logistic regression was carried out to investigate the factors that predicted willingness to discuss HPV in the future (dichotomised for these analyses as ‘not at all’ and ‘not very willing’ versus ‘somewhat’ or ‘very willing’).

## Results

### Sample characteristics

260 health professionals from the UK and Ireland completed the survey (193 online and 67 on paper). It was not possible to calculate a response rate because an accurate denominator could not be calculated. Table 1 shows the sample characteristics. The majority of the sample were female (59.6%, n = 155), had trained in the UK (91.2%, n = 237) and worked mostly in a hospital (97.3%, n = 253). Surgeons were the profession most represented in the sample (36.9%, n = 96) and included head and neck, ear, nose and throat, oral and maxillofacial surgeons. Oncologists were clinical oncologists, who in the UK oversee both chemotherapy and radiotherapy. Some examples of professionals in the 'other' group of health professionals include dieticians, staff nurses, research nurses and radiographers. The internet, medical journals and other colleagues were the top three places health professionals looked for information about HPV (see Table 2).

The median age of the sample was 45 years (range: 21–66). Health professionals had worked in their profession for a median of 17 years (range: 0 to 45 years). Almost all (99.2%) had heard of HPV.

### Experience of discussing HPV with patients

Around 75% had previous experience of discussing HPV with a patient. Surgeons, oncologists and specialist nurses had more experience of telling their patients that their cancer was caused by HPV (X^2^ (12) = 78.49, p < 0.001) and discussing this in detail with their patients (X^2^ (8) = 23.48, p = 0.003) than the speech and language therapists (SLTs) or the ‘other’ group of health professionals. Over two-thirds of oncologists reported initiating the discussion about HPV themselves (67.9%, n = 19), compared with 54.8% (n = 46) of surgeons and 25.7% (n = 9) of specialist nurses (X^2^ (12) = 95.94, p < 0.001). Among SLTs who had discussed HPV with patients (n = 40), 55.0% (n = 22) reported that the patient had initiated the discussion.

### Knowledge of HPV

The proportion of respondents giving the correct answer to each individual knowledge item is shown in Table 3. Across all health professional groups, knowledge of HPV was high. Most knowledge items were correctly answered by over 75% of participants. Two items were answered correctly by less than two-thirds of health professionals: 'HPV usually goes away without needing any treatment' (45.8% correct) and ‘Most sexually active people will get HPV at some point in their lives’ (61.9% correct).

The mean knowledge score was 9.97 out of a possible 12. Total knowledge score was highest in oncologists, followed by surgeons, then specialist nurses, SLTs and the ‘other’ group of health professionals (F (4,246) = 10.48, p < 0.001). Surgeons had a significantly higher mean knowledge score than the ‘other’ group of health professionals (p < 0.001) and SLTs (p < 0.001); and oncologists had a significantly higher mean score than specialist nurses (p = 0.017), SLTs (p < 0.001) and the ‘other’ group of health professionals (p < 0.001). Item by item differences across the professional groups are shown in Table 3.

### Attitudes to discussing HPV

Table 4 shows mean scores by professional group for the five attitude scales. There were significant correlations between many of these variables ranging in strength from 0.13 to 0.58 (see Table 5).

*Confidence in discussing HPV:* The mean (M) score was 3.41 (standard deviation (SD) = 0.83, range 1–5), suggesting overall health professionals were fairly confident about discussing HPV. Confidence talking about HPV varied by professional group (F (4,253) = 22.80, p < 0.001; Table 4) with significantly lower confidence scores for SLTs and health professionals in the ‘other’ group, compared with surgeons (SLTs p < 0.001; ‘other’ p < 0.001), oncologists (SLTs p < 0.001; ‘other’ p < 0.001) and specialist nurses (SLTs p < 0.001; ‘other’ p = 0.036). There was a significant correlation between confidence and number of years practising in their profession (r = 0.138, p = 0.027).

*Negative attitudes to discussing HPV:* In general, negative attitude scores were low (M = 1.93, SD = 0.53) but there was variation across the health professional groups (F (4,252) = 3.49, p = 0.009; Table 4) with significantly higher scores among surgeons, the ‘other’ group of health professionals and SLTs than specialist nurses (surgeons p = 0.012; ‘other’ p = 0.028; SLTs p = 0.014).

*Positive attitudes to discussing HPV:* Conversely, positive attitude scores were high *(*M = 4.22, SD = 0.49) with similar differences across professional groups (F (4,254) = 5.59, p < 0.001; Table 4). Specialist nurses had significantly higher positive attitude scores than surgeons (p < 0.001), oncologists (p = 0.004) and the ‘other’ health professional group (p = 0.022).

*Personal barriers to discussing HPV:* The mean score for personal barriers was 2.87 (SD = 0.69), suggesting some level of disagreement from health professionals about personal barriers to discussing HPV. When comparing professional groups, a significant main effect was found (F (4,253) = 8.89, p < 0.001; Table 4), with surgeons, SLTs and the ‘other’ group reporting significantly higher scores for barriers than specialist nurses (surgeons p = 0.001; SLTs p < 0.001; ‘other’ p = 0.003) and surgeons reporting significantly lower scores for barriers than SLTs (p = 0.04). Personal barriers to discussing HPV were negatively associated with knowledge (r = −0.198, p < 0.01) and positively associated with the need for more information (r = 0.299, p < 0.01).

*Needing more information:* Mean scores were above the mid-point (M = 3.85 and SD = 0.54), suggesting health professionals felt they needed more information about HPV. There were significant differences between health professional groups (F (4,254) = 9.83, p < 0.001; Table 4). SLTs had a significantly greater need for information than surgeons (p < 0.001), oncologists (p < 0.001) and the ‘other’ group of health professionals (p = 0.002).

### Willingness to discuss HPV in the future

Willingness to discuss HPV in the future was significantly positively associated with confidence in discussing HPV (r = 0.582, p < 0.001), positive attitudes to discussing HPV (r = 0.201, p < 0.001) and knowledge (r = 0.347, p < 0.001; Table 5). Significant negative associations were found between willingness to discuss HPV in the future and negative attitudes to discussing HPV (r = −0.232, p < 0.001), personal barriers to discussing HPV (r = −0.492, p < 0.001) and the need for more information (r = −0.149, p < 0.05).

Multivariate logistic regression showed that confidence discussing HPV was a significant predictor of willingness to discuss HPV (adjusted OR = 7.67, 95% CI 1.76–33.44; Table 6), while having personal barriers to discussing HPV was associated with significantly decreased odds of being willing to discuss HPV (OR = 0.13, 95% CI 0.019–0.817). Nagelkerke’s R^2^ indicates that the predictor variables explain 49% of the variance in willingness to discuss HPV in the future.

## Discussion

To our knowledge, this is the first quantitative study assessing knowledge and attitudes about discussing HPV with HNC patients among health professionals in the UK and Ireland. Almost all had heard of HPV and most had looked for information about HPV and HNC. The internet, medical journals and other colleagues were the top three sources of information, supporting findings from previous research [10]. Oncologists had the greatest knowledge, followed by surgeons, specialist nurses, SLTs and the health professionals in the ‘other’ group, suggesting a need to increase knowledge among allied health professionals. The proportion of surgeons answering the specific HNC questions correctly was slightly lower than in a previous study with head and neck surgeons [14], but knowledge was high in both studies.

Supporting previous research about discussing HPV in the context of the HPV vaccination [16], [17], health professionals in this study with greater knowledge also had greater confidence about discussing HPV and were more willing to do so in the future [17]. Factors which had the greatest association with willingness to discuss HPV in the future were confidence and having low personal barriers. Health professionals reporting personal barriers to discussing HPV were less willing to do so, had lower knowledge of HPV and reported needing more information about HPV. This study identifies areas which could be targeted to increase health professionals’ confidence to discuss HPV and also their willingness to do so. Confidence could be increased by improving knowledge, meeting information needs, and addressing personal barriers and negative attitudes towards discussing HPV. This could result in more health professionals discussing HPV with their patients.

Theories of behaviour change aim to explain why people adopt new behaviours and what components may be responsible for that change. The Capability-Opportunity-Motivation Behaviour (COM-B) model [21] is a way of conceptualising the factors needed for a particular behaviour to take place, and could be helpful in understanding the reasons some health professionals do not discuss HPV with their HNC patients, and in identifying targets for interventions aimed at facilitating these discussions. Assessing the results from this study against the components of the COM-B model shows that surgeons and oncologists have the capability, motivation and opportunity to discuss HPV, and that they are doing so. SLTs and other allied health professionals may lack capability because of lower knowledge and confidence; may lack motivation because of their lack of confidence; and may lack opportunity because they are likely to see patients less frequently than surgeons and oncologists.

Knowledge was shown to be highest in surgeons and oncologists, and lowest in SLTs and the ‘other’ group of health professionals. Surgeons and oncologists also showed a greater confidence about discussing HPV than the other health professional groups. SLTs and health professionals in the ‘other’ group reported more personal barriers (e.g. embarrassment) to discussing HPV, and these groups were less willing to discuss HPV. Previous research in dentists has found confidence in performing oral examinations or providing advice about risk habits to be directly correlated with their subsequent performance of oral examinations or provision of advice to patients [22]. Educational materials could be used in future interactions which may help increase knowledge and confidence about having these conversations with patients. Didactic approaches such as information booklets, videos and interactive lectures have been found to be effective at increasing knowledge in dentists [23].

Greater proportions of oncologists, surgeons and specialist nurses than allied health professionals in this sample had told all or most of their patients that their cancer was caused by HPV, had discussed HPV in detail with those patients, and were willing to discuss HPV in the future. These health professionals are ideally situated to have this discussion with patients as they are likely to see patients more frequently than allied health professionals. As there was still a proportion of allied health professionals who had discussed HPV in detail with these patients, this demonstrates that it is possible for questions to come up at any point during patients’ diagnosis, treatment and aftercare, consistent with previous findings [24]. It is therefore important that all health professionals caring for these patients feel adequately informed and motivated, and have the necessary skills to have these discussions with patients. Further training could be targeted at increasing these three components in these health professional groups to help facilitate the discussions of HPV with patients. It is important for health professionals to have conversations with their patients about HPV so that information can be delivered by a trustworthy and reliable source, who can also address concerns and uncertainties around the sexually transmitted nature of HPV.

This study has some limitations. Due to the recruitment methods used, it was not possible to calculate a response rate and therefore it is difficult to assess the representativeness of the sample. The associations and differences found in this sample are more likely to be generalisable than the prevalence estimates. The sample was also self-selected and it is possible that those who were most interested in HPV were those who took part. Further research would also be needed to determine whether these results extend to other countries, beyond the UK and Ireland. As some of the participants completed the survey at study days, it is possible that some of them completed it following the talks given that day and this could have influenced their responses, perhaps leading to inflated estimates of knowledge. Although some of the questions assessing participants’ knowledge were from a validated scale, not all of the questions were taken from validated scales. Future work should look at validating a measure assessing knowledge of HPV-OSCC.

## Conclusion

This study shows that knowledge and attitudes about HPV-OSCC varies across health professional groups, with surgeons, oncologists and specialist nurses having more experience of discussing HPV-OSCC with their patients. Behaviour change models could provide useful frameworks for future training and interventions to equip all health professionals with the vital components to be able to discuss HPV-OSCC with their patients and increase their willingness to do so.

## Author’s contributions

RD, LM and JW conceived of the study and developed the questionnaire. RD was responsible for data collection. Data analysis was done by RD, with input from AF, LM and JW. RD was responsible for the interpretation of the analyses and write-up of the results, with input from AF, LM and JW. All authors have seen and approved the final version of the manuscript before submission.

## Conflict of interest

None declared.

## Role of funder

Funders have had no role in the study design, the collection, analysis, interpretation of data, the writing of the article or the decision to submit it for publication.

## Figures and Tables

**Table 1:** Sample characteristics.

Characteristic	Number of participants (n = 260)
Age [median(range)]	45 (21–66)

Sex [n(%)]	
Male	105 (40.4)
Female	155 (59.6)

Profession [n(%)]	
Surgeon	96 (36.9)
Oncologist	28 (10.8)
Specialist nurse	40 (15.4)
Speech and language therapist	59 (22.7)
Other^a^	37 (14.2)

Years in profession [median(range)]	17 (0–45)

Trained in the UK [n(%)]	
Yes	237 (91.2)
No	23 (8.8)

Main place of work [n(%)]	
Hospital	253 (97.3)
Hospice	1 (0.4)
Rehabilitation Centre	1 (0.4)
Other	5 (1.9)

Have you ever heard of HPV [n(%)]	
Yes	258 (99.2)
No	1 (0.4)
Don't know	1 (0.4)

Looked for information on HPV and HNC [n(%)]	
Yes	245 (94.2)
No	14 (5.4)
Don't remember	1 (0.4)

Sources searched for information on HPV and HNC [n(%)]	
Internet	205 (78.8)
Medical Journals	191 (73.5)
Other colleagues	183 (70.4)
Conferences	167 (64.2)
Professional organisations	78 (30.0)
Media	53 (20.4)
Study information leaflets	1 (0.4)

Willingness to discuss HPV with patients in the future [n(%)]	
Surgeons	86 (91.5)
Oncologists	26 (92.9)
Specialist nurses	35 (87.5)
Speech and language therapists	43 (72.9)
Other	25 (69.4)

a Includes dieticians, radiographers, staff nurses, research nurses.

**Table 2:** Percentage of each professional group consulting different sources of information about HPV and HNC.

	Overall (n = 260)	Surgeons (n = 96)	Oncologists (n = 28)	Specialist nurses (n = 40)	Speech and language therapists (n = 59)	Other (n = 37)
Internet	79%	85%	86%	83%	69%	68%
Medical Journals	74%	86%	96%	63%	59%	57%
Other colleagues	70%	71%	75%	83%	66%	59%
Conferences	64%	80%	82%	45%	54%	46%
Professional organisations	30%	40%	36%	33%	19%	16%
Media	20%	27%	18%	23%	14%	14%
Study information leaflets	0.4%	0	0	0	0	3%

NB: Columns do not total 100% because health professionals could consult multiple sources of information.

**Table 3:** Correct responses (n/%) to individual knowledge of HPV items among all professional groups (n = 260).*

	Overall	Surgeons	Oncologists	Specialist nurses	Speech and language therapists	Other	X^2^ (p value)
		(n = 96)	(n = 28)	(n = 40)	(n = 59)	(n = 37)	
HPV often has no visible signs or symptoms	239 (91.9)	91 (94.8)	28 (100)	38 (95.0)	49 (83.1)	33 (89.2)	**10.66 (0.031)**
HPV is very rare (F)	244 (93.8)	94 (97.9)	28 (100)	38 (95.0)	52 (88.1)	32 (86.5)	**11.48 (0.022)**
A person could have HPV for many years without knowing it	253 (97.7)	95 (99.0)	27 (96.4)	38 (97.4)	56 (94.9)	37 (100)	3.77 (0.438)
Having many sexual partners increases the risk of getting HPV	230 (88.8)	91 (94.8)	28 (100)	30 (76.9)	49 (83.1)	32 (86.5)	**14.69 (0.005)**
HPV can cause cervical cancer	253 (97.3)	96 (100)	28 (100)	39 (97.5)	55 (93.2)	35 (94.6)	8.24 (0.083)
HPV usually goes away without needing any treatment	119 (45.8)	47 (49.0)	17 (60.7)	18 (45.0)	25 (42.4)	12 (32.4)	5.85 (0.211)
Most sexually active people will get HPV at some point in their lives	159 (61.9)	62 (65.3)	21 (75.0)	25 (65.8)	31 (52.5)	20 (54.1)	5.89 (0.207)
HPV can cause oral cancer	240 (92.7)	85 (88.5)	27 (96.4)	38 (97.4)	53 (89.8)	37 (100)	7.92 (0.095)
The oral tongue is the principal head and neck cancer site associated with HPV (F)	191 (93.7)	83 (86.5)	26 (92.9)	24 (60.0)	39 (66.1)	19 (52.8)	**27.15 (**<**0.001)**
HPV is a relatively uncommon sexually transmitted infection (F)	201 (78.2)	84 (87.5)	27 (96.4)	26 (68.4)	40 (67.8)	24 (66.7)	**19.02 (0.001)**
HPV is associated with a much improved prognosis for patients with head and neck cancer	229 (88.1)	87 (90.6)	27 (96.4)	34 (85.0)	53 (89.8)	28 (75.7)	8.41 (0.078)
Most patients with oral HPV experience symptoms of the infection (F)	223 (85.8)	91 (94.8)	27 (96.4)	34 (85.0)	42 (71.2)	29 (78.4)	**20.96 (**<**0.001)**
							ANOVA
Total knowledge score: mean (standard deviation)	9.97 (1.82)	10.48^ab^ (1.32)	11.11^cde^ (0.83)	9.74 (1.85)^c^	9.22 (2.06)^bd^	9.14 (2.2)^ae^	F (4,246) = 10.48, p < 0.001

* n varies slightly between items due to missing data; (F) indicates items for which ‘false’ is the correct response. All other items are true; ^abcde^ indicates which groups are significantly different from each other in post hoc tests.

**Table 4:** Attitudes to discussing HPV with oropharyngeal cancer patients: mean scale scores across health professional groups.

	Surgeons	Oncologists	Specialist nurses	Speech and language therapists	Other	F (p value)
	(n = 96)	(n = 28)	(n = 40)	(n = 59)	(n = 37)	
Confidence talking about HPV	3.74 (0.65)^ab^	3.88 (0.58)^cd^	3.59 (0.74)^ef^	2.74 (0.82)^ade^	3.10 (0.80)^bcf^	22.80 (<0.001)
Negative attitudes to discussing HPV	1.98 (0.53)^a^	1.90 (0.47)	1.66 (0.55)^abc^	2.00 (0.42)^c^	2.02 (0.60)^b^	3.49 (0.009)
Positive attitudes to discussing HPV	4.12 (0.50)^a^	4.10 (0.46)^b^	4.52 (0.48)^abc^	4.26 (0.42)	4.18 (0.48)^c^	5.59 (<0.001)
Personal barriers to discussing HPV	2.86 (0.66)^ab^	2.80 (0.69)	2.38 (0.66)^acd^	3.17 (0.64)^bd^	2.93 (0.61)^c^	8.89 (<0.001)
Need for more information	3.69 (0.52)^a^	3.66 (0.60)^b^	3.95 (0.38)	4.18 (0.41)^abc^	3.77 (0.64)^c^	9.83 (<0.001)

n for items varies slightly due to missing data.

^abcdef^indicates across rows which groups are significantly different from each other in post hoc tests.

Scores range from 1 to 5; 1 represents low confidence, less negative attitudes, less positive attitudes, fewer personal barriers and less need for information.

**Table 5:** Correlation matrix for all attitudinal factors, total knowledge score and willingness to discuss HPV.

	Confidence in discussing HPV	Negative attitudes to discussing HPV	Positive attitudes to discussing HPV	Personal barriers to discussing HPV	Need for more information	Knowledge	Willingness to discuss HPV
Confidence in discussing HPV	1						
Negative attitudes to discussing HPV	−0.097	1					
Positive attitudes to discussing HPV	0.132*	−0.508**	1				
Personal barriers to discussing HPV	−0.553**	0.231**	−0.63**	1			
Need for more information	−0.415**	−0.213**	0.156*	0.299**	1		
Knowledge	0.435**	−0.071	−0.033	−0.198**	−0.154*	1	
Willingness to discuss HPV	0.582**	−0.232**	0.201**	−0.492**	−0.149*	0.347**	1

**Table 6:** Unadjusted and adjusted odds ratios (OR) for factors predicting willingness to discuss HPV in the future.

	Unadjusted OR (95% CI)	Sig. (p value)	Adjusted* OR (95% CI)	Sig. (p value)
Confidence in discussing HPV	8.34 (3.24–21.45)	<0.001	7.67 (1.76–33.44)	0.007
Personal barriers	0.06 (0.01–0.24)	<0.001	0.13 (0.02–0.82)	0.03
Negative attitudes to discussing HPV	0.40 (0.11–1.41)	0.153	0.34 (0.04–2.84)	0.322
Positive attitudes to discussing HPV	1.86 (0.52–6.75)	0.342	0.25 (0.02–2.82)	0.263
More information	0.53 (0.15–1.94)	0.34	5.40 (0.45–64.24)	0.182
