# Supplementary material for: Discussing HPV with oropharyngeal cancer patients: A cross-sectional survey of attitudes in health professionals
Source: Oral Oncol. 2017 May;68:67–73. doi: 10.1016/j.oraloncology.2017.03.014 (PMC5414413; doi:10.1016/j.oraloncology.2017.03.014)
Supplement: Supplementary data 1 [file mmc1.docx]

**Supplementary file: Survey
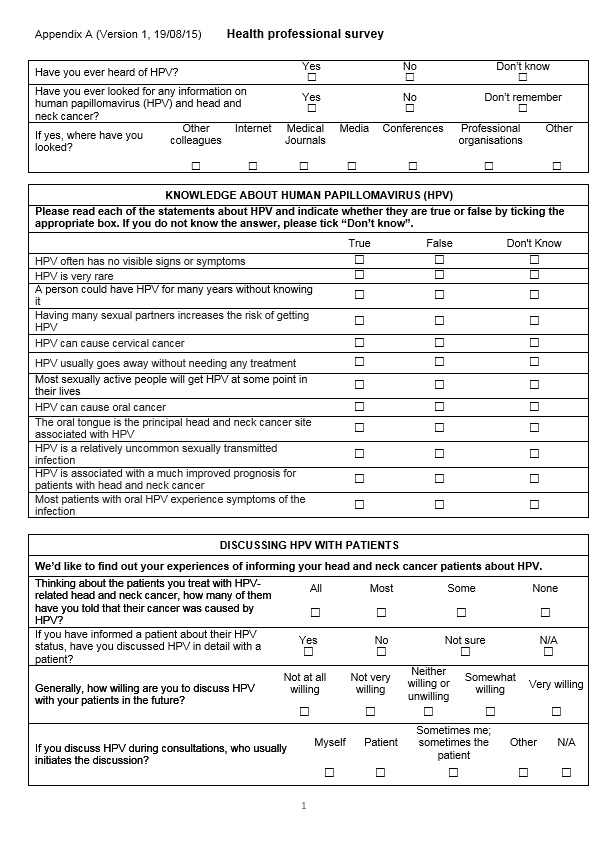
**

**
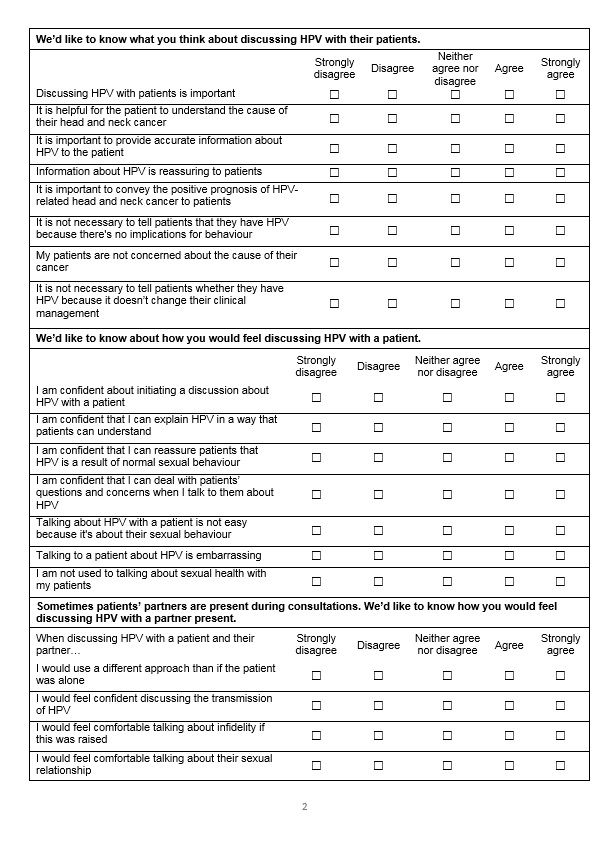
**

**
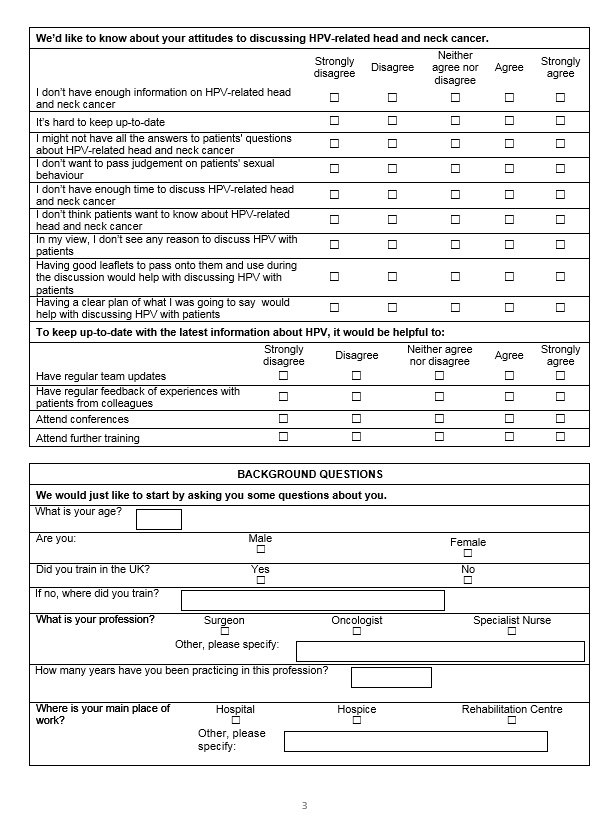
**

**Supplementary file: Details of the sources of recruitment**

| Recruitment source | Details about the source | Which health professionals | Online/paper |
| --- | --- | --- | --- |
| NHS Cancer networks (e.g. London Cancer Alliance) | Strategic Clinical Networks focus on priority service areas to bring about improvement in the quality and equity of care and outcomes of their population, both now and in the future. There are 14 cancer networks across England and Wales. Three of the 14 networks sent out information and the link to the survey. | All disciplines | Online |
| National Cancer Research Institute | The National Cancer Research Institute is a UK-wide partnership between research funders working together to make faster progress against cancer. | All disciplines | Online |
| National Cancer Intelligence Network (NCIN) | **The NCIN is a UK-wide initiative, working to drive improvements in standards of cancer care and clinical outcomes by improving and using the information collected about cancer patients for analysis, publication and research.** | All disciplines | Online |
| Head and Neck Cancer Masterclass | This masterclass offered the opportunity for all professional groups working with head & neck cancer patients to come together to hear about the latest developments in treatment and care and to discuss issues of mutual interest. The meeting was attended by around 80 – 100 health professionals from across the multi-disciplinary team. | Predominantly Clinical Nurse Specialists, Speech & Language Therapists, Radiographers and Dietitians | Paper |
| Joint Meeting of the Northern and Southern Clinical Excellence Networks for Head and Neck Cancer | The purpose of this joint study day was to enhance participants’ knowledge of HPV in the context of head and neck cancer, critical issues in the management of acute and late swallowing toxicities and clinical trials aiming to ascertain optimal treatment delivery aimed at minimising late dysphagia. | Predominantly Clinical Nurse Specialists, Speech & Language Therapists, Radiographers and Dietitians | Paper |
| Head and Neck Study Day – The Royal Marsden Hospital | This meeting explored the issues surrounding cutaneous malignancy of the head and neck. It included: evidence-based lectures that focused on the management of a variety of skin and adnexal cancers including non-invasive diagnostic testing, the assessment of regional nodes, the role of adjuvant treatment and targeted therapies, a discussion on how surgeon-level reporting might influence the delivery of head and neck cancer care. | Specialist Registrars and Consultants in Head and Neck / Maxillofacial / ENT / Plastic Surgery, Dermatologists, Clinical and Medical Oncologists, Pathologists and all members of the extended Head and Neck Team | Paper |
| British Association of Head and Neck Oncologists Annual Scientific Meeting 2015 | Email addresses were searched for online for the names on the delegate lists. | Clinical oncologists, surgeons, clinical nurse specialists, maxillofacial oncologists, ENT | Online |
| British Association of Oral and Maxillofacial Surgeons – list of hospital units | Email addresses were searched for online for the names on the delegate lists. | Oral and Maxillofacial Surgeons | Online |
| A list of contacts from a researcher in Ireland | A researcher who works closely with my research group provided a list of health professionals who had been involved in previous research with them and were happy to be involved in future research. All health professionals were emailed. | Clinical oncologists, surgeons, clinical nurse specialists, maxillofacial oncologists, ENT | Online |
| Multidisciplinary teams (MDT) | Some health professionals distributed it to their hospital MDT | All disciplines | Online |
| British Association of Head and Neck Oncology Nurses website | The study was advertised on the website | Head and neck cancer oncology nurses | Online |

| Item | Rotated factor loadings | Eigenvalue  (% of variance) | α |
| --- | --- | --- | --- |
| **Confidence in discussing HPV** | | 5.87 (23.5) | 0.89 |
| I am confident that I can deal with patients’ questions and concerns when I talk to them about HPV | 0.845 |  |  |
| I am confident that I can explain HPV in a way that patients can understand | 0.836 |  |  |
| I am confident about initiating a discussion about HPV with a patient | 0.828 |  |  |
| I would feel confident discussing the transmission of HPV | 0.747 |  |  |
| I don’t have enough information on HPV-related head and neck cancer | -0.576 |  |  |
| **Personal barriers to discussing HPV** | | 4.14 (16.6) | 0.78 |
| I would feel comfortable talking about their sexual relationship* | -0.707 |  |  |
| Talking about HPV with a patient is not easy because it's about their sexual behaviour | 0.664 |  |  |
| I would feel comfortable talking about infidelity if this was raised* | -0.647 |  |  |
| Talking to a patient about HPV is embarrassing | 0.621 |  |  |
| I don’t have enough time to discuss HPV-related head and neck cancer | 0.555 |  |  |
| I am not used to talking about sexual health with my patients | 0.494 |  |  |
| **Negative attitudes to discussing HPV** | | 1.60 (6.4) | 0.75 |
| I don’t see any reason to discuss HPV with patients | 0.735 |  |  |
| I don’t think patients want to know about HPV-related head and neck cancer | 0.732 |  |  |
| It is not necessary to tell patients whether they have HPV because it doesn’t change their clinical management | 0.694 |  |  |
| My patients are not concerned about the cause of their cancer | 0.659 |  |  |
| It is not necessary to tell patients that they have HPV because there's no implications for behaviour | 0.535 |  |  |
| **Positive attitudes to discussing HPV** | | 1.52 (6.1) | 0.76 |
| It is helpful for the patient to understand the cause of their head and neck cancer | 0.694 |  |  |
| Information about HPV is reassuring to patients | 0.683 |  |  |
| It is important to convey the positive prognosis of HPV-related head and neck cancer to patients | 0.657 |  |  |
| Discussing HPV with patients is important | 0.616 |  |  |
| It is important to provide accurate information about HPV to the patient | 0.582 |  |  |
| **Need for more information** |  | 1.34 (5.4) | 0.64 |
| HPV-related head and neck cancer is an evolving area so it’s hard to keep up-to-date | 0.687 |  |  |
| I might not have all the answers to patients' questions about HPV-related head and neck cancer | 0.614 |  |  |
| Having a clear plan of what I was going to say would help me discuss HPV with patients | 0.599 |  |  |
| Having a leaflet to pass onto patients would help me discuss HPV with them | 0.497 |  |  |
| *these items were reversed |  |  |  |

Supplementary file: Summary of PCA results for the survey (n=254)
